# Supplementary material for: The Systems Biology Research Tool: evolvable open-source software
Source: BMC Syst Biol. 2008 Jun 29;2:55. doi: 10.1186/1752-0509-2-55 (PMC2446383; doi:10.1186/1752-0509-2-55)
Supplement: Additional file 1 — SBRT Archive. An archive of the current version of the Systems Biology Research Tool. [file 1752-0509-2-55-S1.zip › sbrt-1.4.0/doc/developers_guide/api/sbrt/shell/mng/package-summary.html]

sbrt.shell.mng


|  |  |  |
| --- | --- | --- |
| **Overview** \| **Package** \| Class \| **Use** \| **Tree** \| **Deprecated** \| **Index** \| **Help** | |  |
| **PREV PACKAGE**   **NEXT PACKAGE** | **FRAMES**    **NO FRAMES**     **All Classes** |  |


---

## Package sbrt.shell.mng

Provides classes and interfaces for creating process managers.

**See:**
  
          **Description**

| **Interface Summary** | |
| --- | --- |
| **KernelProcessManager<K,V>** | This interface is used to represent process managers. |
| **ManagerInput<K,V>** | This interface is used to represent the input to `KernelProcessManager` objects. |
| **ProcessManager<K,V>** | This interface is used to represent process managers. |
| **ShellProcessManager<K,V>** | This interface is used to represent shell process managers. |

| **Class Summary** | |
| --- | --- |
| **AbstractProcessManager** | This class is a skeleton implemenation of `KernelProcessManager`. |
| **MapManagerInput<K,V>** | This class is a concrete implemenation of `ManagerInput`. |

## Package sbrt.shell.mng Description

Provides classes and interfaces for creating process managers.

---


|  |  |  |
| --- | --- | --- |
| **Overview** \| **Package** \| Class \| **Use** \| **Tree** \| **Deprecated** \| **Index** \| **Help** | |  |
| **PREV PACKAGE**   **NEXT PACKAGE** | **FRAMES**    **NO FRAMES**     **All Classes** |  |


---
